# Urinary α-Carboxyethyl Hydroxychromanol (α-CEHC)—A Biomarker of Vitamin E Intake: A Scoping Review

**DOI:** 10.1016/j.tjnut.2026.101486

**Published:** 2026-03-21

**Authors:** Yochana Benchetrit, Maret G Traber

**Affiliations:** 1 Faculty of Medicine, University of Toronto, Toronto, ON, Canada; 2 Linus Pauling Institute, Oregon State University, Corvallis, OR, United States

**Keywords:** CEHC, α-CEHC, tocochromanol catabolism, vitamin E, urinary biomarkers

## Abstract

Urinary α-carboxyethyl hydroxychromanol (α-CEHC), the terminal catabolic product of α-tocopherol, has been proposed as a noninvasive biomarker of vitamin E intake. Existing human studies vary substantially in design, population characteristics, analytical methods, and reporting practices, limiting the comparability of findings across the literature. The objective of this scoping review is to systematically map all human evidence evaluating urinary α-CEHC in relation to vitamin E intake, catabolism, or health status. The protocol for this scoping review was registered in Open Science Framework on 26 October 2025 (https://osf.io/a7gkv). Following Joanna Briggs Institute (JBI) methodology and PRISMA-ScR reporting standards, we searched MEDLINE, EMBASE, Web of Science, CENTRAL, and CINAHL. The initial literature search yielded 586 records. Two reviewers independently screened all records, extracted data using piloted forms, and organized findings into thematic domains reflecting biomarker responsiveness, methodological variability, and clinical contexts. A total of 34 studies were included, representing a wide range of vitamin E interventions, urine collection approaches, and analytical methods. Study heterogeneity was substantial. The increases in urinary α-CEHC excretion in response to supplemental vitamin E dose reported in 10 intervention studies were highly correlated (*R*^2^ = 0.7819, *P* < 0.0001). Overall, urinary α-CEHC appears to be a promising integrated measure of vitamin E catabolism; however, methodological inconsistencies across studies limit the ability to compare results between individuals on routine diets. Standardized urine collection protocols, conjugate handling procedures, normalization strategies, and analytical techniques will be necessary before α-CEHC can be validated for clinical application.

This study was registered in Open Science Framework on 26 October, 2025 (https://osf.io/a7gkv) as a scoping review.

## Introduction

Tocochromanols are a family of 8 naturally occurring compounds (4 tocopherols and 4 tocotrienols) which function as lipid-soluble antioxidants that protect plant and animal cell membranes from oxidative damage [1]. Among these, α-tocopherol is the most biologically active form in humans and the only form recognized as a vitamin [2]. Assessment of α-tocopherol (vitamin E) status has traditionally relied on measuring plasma or tissue α-tocopherol concentrations; however, these values are strongly influenced by circulating lipid levels [3] and disorders of lipoprotein transport or fat absorption, such as abetalipoproteinemia (ABL), cystic fibrosis, or cholestasis, limiting their validity as independent indicators of vitamin E sufficiency [4,5]. Because CEHC metabolites are rapidly cleared from the circulation by renal excretion, plasma concentrations represent only a transient pool, whereas urinary measurements integrate the cumulative formation and elimination of these metabolites over time [6].

**FIGURE 1:**
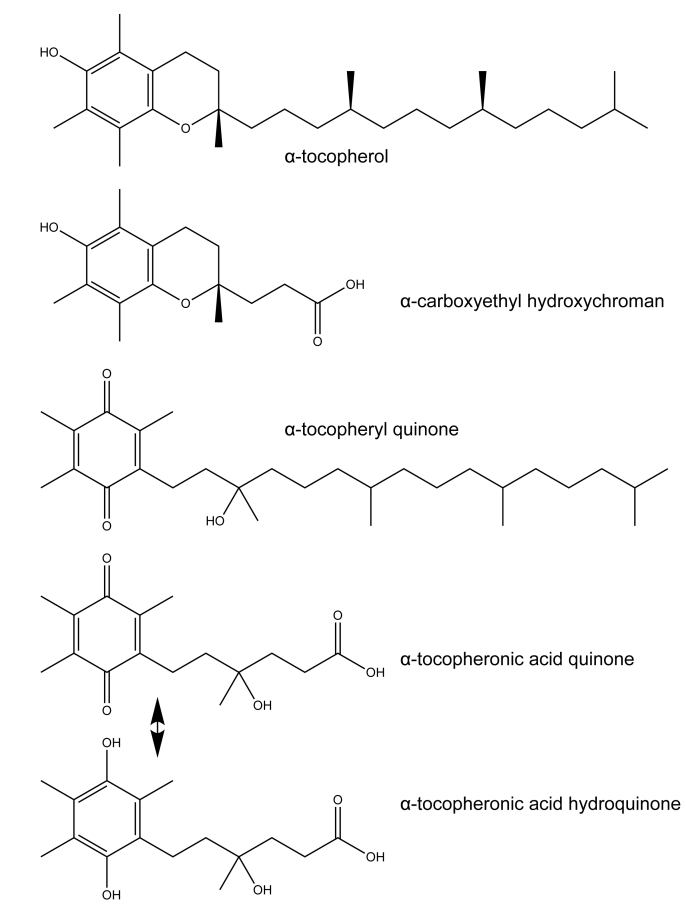
Tocochromanol structures. Shown are α-tocopherol; α-carboxyethyl hydroxychromanol (α-CEHC); its catabolite; α-tocopheryl quinone (the 2-electron oxidation product of α-tocopherol); and its catabolite α-tocopheronolactone hydroquinone (α-TLHQ) in equilibrium with α-tocopheronic acid (α-TLQ), also known as Simon’s metabolite [7,8].

The first descriptions of vitamin E metabolism in humans using radioactive α-tocopherol were reported in the 1950s—the Simon metabolite was characterized tentatively as 2-(3-hydroxy-3-methyl-5-carboxypentyl)-3,5,6-trimethylbenzoquinone and its γ-lactone [7,8]. Early investigations into vitamin E metabolism focused on identifying these tocopherol metabolites and characterizing the ω-oxidation steps and β-oxidation pathway. Catabolism of tocochromanols, including “excess” α-tocopherol, is a major mechanism to quickly rid the body of these molecules in a xenobiotic process [[9], [10], [11], [12]]. The cytochrome P450 4F2 (CYP4F2) ω-hydroxylates the sidechain of tocochromanols to form the long chain, 13’-hydroxy catabolite [10,13]. The catabolite undergoes oxidation to form the 13’-carboxy catabolite, which then undergoes several rounds of β-oxidation [14] to form carboxyethyl hydroxychromanol (CEHC) (Figure 1 [7,8]). CYP4F2 is the only enzyme shown to exhibit tocochromanol-ω-hydroxylase activity in humans. Two common variants (V433M and W12G) alter CYP4F2 activity, but it is unclear how these variants affect overall vitamin E status [15]. CYP4F2 has also been shown to hydroxylate tocopheryl quinones [16].

Several studies in the late 1980s and early 1990s described putative terminal metabolites using qualitative or semiquantitative approaches, including oxidized chromanol products. These early studies were excluded from the present review because the methods were too insensitive to provide quantitative urinary α-CEHC measurements, although the higher γ-CEHC concentrations could be detected. The identification of α-CEHC as the terminal water-soluble metabolite of α-tocopherol and its reproducible quantification in urine marked a turning point in the field. For example, Schultz et al. [17] reported using GC-MS to detect urinary α-CEHC in participants who consumed a supplement of 50–150 mg *RRR*-α-tocopherol per day for 7 d. Traber et al. [18] reported using GC-MS data from 6 individuals who consumed a single encapsulated dose of 150 mg each *RRR*-α-[5-(C^2^H_3_)]- and *all racemic (all-rac)*-α-[5,7(C^2^H_3_)_2_]-tocopheryl acetates (d_3_- and d_6_-α-tocopheryl acetates). These studies were among the first to demonstrate measurable urinary α-CEHC after supplemental α-tocopherol intake in humans, establishing urinary excretion as a quantifiable outcome. Subsequent studies built upon this framework using increasingly refined analytical techniques, forming the basis for the studies synthesized in the present review.

Despite over 2 decades of research on CEHC catabolites, human studies differ widely in design, population type, vitamin E form (natural compared with synthetic; α- compared with other tocochromanol-enriched mixtures), and analytical methods. Our review aims to *1*) collate and characterize the breadth of evidence on urinary α-CEHC, *2*) identify methodological inconsistencies that limit cross-study comparability, and *3*) delineate knowledge gaps before formal diagnostic accuracy or meta-analytic work can be pursued. To address these gaps, we conducted a scoping review. The primary objective of this review was to map all available human evidence evaluating urinary α-CEHC as a biomarker of vitamin E intake. In this review, we *1*) catalog urine collection, normalization, and analytical methods used across studies to characterize methodological variability; *2*) synthesize evidence on the responsiveness of urinary α-CEHC to vitamin E intake or supplementation; and *3*) describe the clinical and physiological contexts in which urinary α-CEHC has been measured.

## Methods

### Protocol and registration

This review was conducted according to the Joanna Briggs Institute (JBI) guidelines for scoping reviews and is reported using the PRISMA-ScR framework [19,20]. The protocol for this study was registered in Open Science Framework on 26 October, 2025 (https://osf.io/a7gkv) as a scoping review.

### Eligibility criteria

Studies were eligible if they involved humans of any age, sex, or health status and provided quantitative or semiquantitative urinary measurements of α-CEHC in the context of vitamin E intake, supplementation, deficiency, or metabolic activity. Eligible study designs included randomized and nonrandomized trials, controlled supplementation studies, tracer studies, cross-sectional observations, and case reports with extractable urinary α-CEHC data. Studies were excluded if they involved only animal or in vitro data, if they reported tocochromanol concentrations without urinary α-CEHC catabolites, if they reported duplicate information, or were reviews, commentaries, or conference abstracts without extractable quantitative data related to urinary α-CEHC formation.

### Search strategy

The literature search was performed on 26 October 2025. Searches were conducted in MEDLINE (Ovid), EMBASE (Ovid), Web of Science Core Collection, Cochrane CENTRAL, and CINAHL (EBSCOhost), without restrictions on date, language, or publication type. The search strategy was developed by lead author YB in consultation with a university evidence synthesis librarian, with the full search strategy provided in Supplementary Methods 1. The strategy incorporated controlled vocabulary and free-text terms related to vitamin E, tocopherols, CEHC catabolites, and urinary excretion.

### Study screening and extraction

All records were imported into Covidence (Veritas Health Innovation Ltd) for deduplication and screening. Using the inclusion and exclusion criteria, YB and MT independently completed title and abstract screening in Covidence after calibrating on a sample of 5 records. Studies referencing CEHC terminology in titles, abstracts, keywords, or main text were considered for full-text review, and the lead authors independently completed the full-text screening in Covidence. Reasons for exclusion at the full-text stage were documented within Covidence, and disagreements were resolved by discussion until a consensus was reached. In the full-text screening stage, studies were included to proceed to extraction only if they had quantitative urinary α-CEHC measurements.

Data extraction was performed using a structured and piloted extraction form within Covidence. Extracted variables included bibliographic details (author, year, journal, country, funding), study design and setting, sample size and demographics, participant health status, tocochromanol supplement dose and form (e.g., *RRR*-α-tocopherol, *all-rac*-α-tocopherol, γ-rich or tocotrienol-rich mixtures), route and duration of exposure, urine collection protocols (e.g., spot, timed, or 24-h urine collections), normalization methods (e.g., creatinine or total volume), storage and handling conditions, analytical techniques, calibration procedures, conjugate handling methods, limits of detection or quantification when available, and all outcome measures related to urinary α-CEHC catabolites.

### Data synthesis

Quantitative data related to urinary α-CEHC was extracted from Covidence and synthesized using Excel (Microsoft). Using the molar mass of creatinine, units were standardized to μmol/mol creatinine if data were reported as α-CEHC per creatinine, with original units retained for transparency. Some studies only reported urinary α-CEHC values in a graph; in this case, we did their best to estimate both the mean and variance measures by visual inspection. For studies reporting daily excretion, values were approximated using the assumption that 1 d corresponds to 1 g creatinine excretion. All standardization calculations are provided in Supplementary Data 1. For studies in which stable isotope-labeled α-tocopherol was administered, the total urinary α-CEHC excretion postsupplementation is reported as a sum of unlabeled plus labeled α-CEHCs. All standardization calculations are provided in Supplementary Data 1. Tables and a descriptive plot were used to summarize study characteristics and outcomes. The plot and Pearson’s correlation were made using Prism 10 for MacOS (GraphPad Software, graphpad.com).

## Results

### Study selection

**FIGURE 2:**
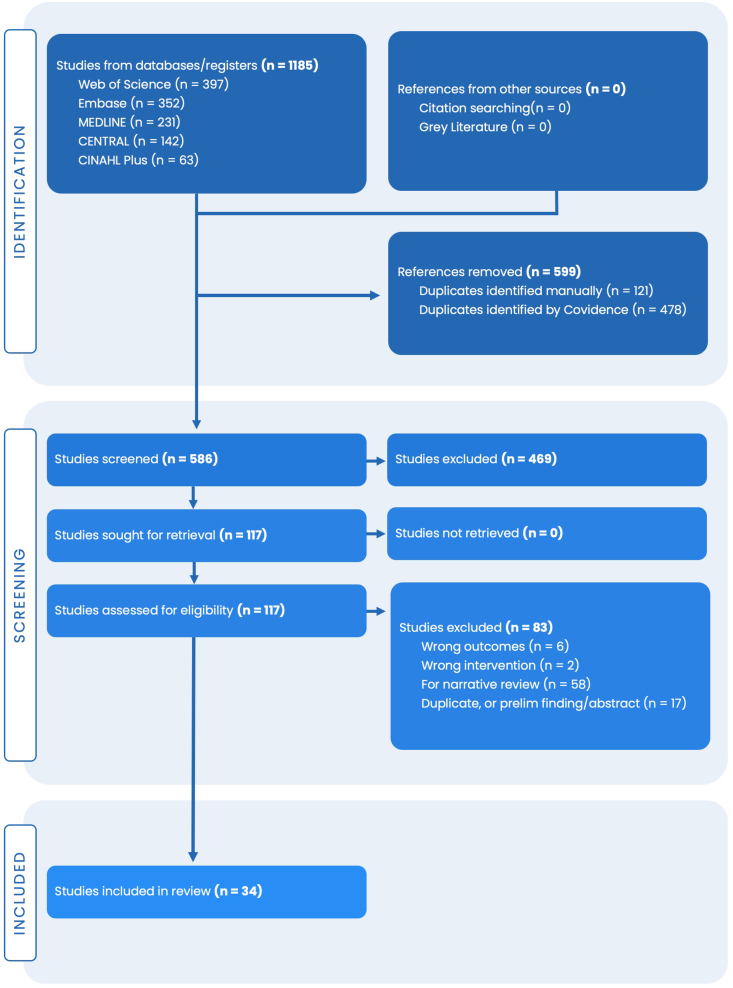
PRISMA 2020 flow diagram. The study selection process is summarized in the PRISMA 2020 flow diagram. The diagram shows how the initial literature search, which yielded 586 records, was culled to 34 studies that met the inclusion criteria and were included in the final synthesis.

The initial literature search yielded 586 records from MEDLINE, EMBASE, Web of Science, CENTRAL, and CINAHL databases. After manual removal of additional duplicates, 469 studies were screened for eligibility by title and abstract. Of these, 352 studies were excluded due to absence of urinary α-CEHC measurements, nonhuman study populations, or review-only publications without extractable data. The full texts of the remaining 117 articles were assessed for eligibility, and 83 were excluded as they did not meet the defined inclusion criteria. Ultimately, 34 studies met the inclusion criteria and were included in the final synthesis. The study selection process is summarized in the PRISMA 2020 flow diagram (Figure 2).

### Study characteristics

The 34 included studies span >2 decades of research on vitamin E catabolism and urinary α-CEHC excretion, ranging from early metabolic investigations in the mid-1990s to more recent intervention and clinical studies. Key study characteristics are summarized in Table 1 [6,17,18,[21], [22], [23], [24], [25], [26], [27], [28], [29], [30], [31], [32], [33], [34], [35], [36], [37], [38], [39], [40], [41], [42], [43], [44], [45], [46], [47], [48], [49], [50], [51]]. Study designs were heterogeneous and included stable isotope tracer studies [6,18,[31], [32], [33], [34], [35], [36]], randomized controlled trials [[26], [27], [28], [29], [30]], crossover studies [[37], [38], [39]], cross-sectional observational studies, controlled supplementation studies, a phase I supplementation/pharmacokinetic study, controlled supplementation with cross-sectional observation, and analytical validation/method development studies. The stable isotope tracer studies included those with single-arm [31,33,35], parallel group [32], randomized crossover design [34,36], controlled supplementation [18], and controlled supplementation with crossover design [6]. Crossover studies included both randomized crossover designs [37,38] and nonrandomized controlled supplementation [39]. Controlled supplementation studies included those with single-arm, parallel group, repeated measures, and withdrawal/repletion designs, with 2 studies having both a controlled supplementation and cross-sectional observational design [46,47]. Two analytical validation studies used either stable isotope-labeled tocopherols [48] or cross-sectional clinical application [49] as proof-of-concept applications but were classified separately because their primary objective was assay development rather than biological inference.

**TABLE 1:** Characteristics of the studies included in the scoping review (*N* = 34)

Characteristic	Number of studies	References
Study design		
Cross-sectional observational study	5	[[21], [22], [23], [24], [25]]
Randomized controlled trial	5	[[26], [27], [28], [29], [30]]
Stable isotope study	8	[6,18,[31], [32], [33], [34], [35], [36]]
Crossover study	3	[[37], [38], [39]]
Controlled supplementation study (nonrandomized, repeated measures)	7	[[17], [40], [41], [42], [43], [44], [45]]
Controlled supplementation + cross-sectional observational	2	[46,47]
Analytical validation	3	[[48], [49], [50]]
Phase I supplementation/pharmacokinetic study	1	[51]
Population		
Healthy only	22	[6,17,18,[21], [22], [23], [24],^27^,^28^,[31], [32], [33], [34],^36^,^37^,[39], [40], [41],^43^,^46^,^49^,^50^]
Disease state only (metabolic syndrome, diabetes, etc.)	6	[21,[26], [27], [28], [29],^36^]
Mixed (healthy and disease state)	3	[25,42,49]
Smokers only	1	[30]
Smokers vs. nonsmokers	2	[22,32]
Reported quantitative unit standardization		
Creatinine	19	[6,[21], [22], [23], [24], [25], [26], [27], [28],^30^,[32], [33], [34], [35], [36],^40^,^45^,^47^,^49^]
Excreted per unit of time	7	[17,18,39,42,43,46,50]
Volume	4	[29,44,48,51]
Other: not reported, percentage of baseline, cumulative amount, etc.	4	[31,37,38,41]
Urine collection method		
24-h urine collection	18	[[17], [18], [21], [22], [23], [24], [26], [27], [28], [31], [32], [33], [40], [41], [42], [43], [46], [50]]
Serial pooled collections (i.e., subdivided: 0–6 h, 6–12 h, 12–24 h, 24–48 h)	5	[6,34,36,37,39]
Single time point (spot sample) collection(s)	8	[[25], [29], [38], [44], [45], [47], [49], [51]]
Not applicable (analytical matrix; single unspecified time point)	1	[49]
Combination of 24-h urine and spot sample collections	2	[35,48]
Analytical method		
GC-MS	11	[17,18,25,27,31,32,35,38,42,47,48]
HPLC-ECD	10	[22,24,29,39,41,43,44,46,50,51]
LC-MS	6	[23,28,30,33,34,37]
UPLC-MS/MS	7	[6,21,26,36,40,45,49]
Country		
United States	15	[6,23,26,[28], [29], [30],^32^,^34^,^36^,^40^,^41^,^45^,^50^,^51^]
Germany	5	[17,18,31,37,42]
Italy	2	[35,48]
United Kingdom	4	[22,38,47,49]
Japan	4	[39,43,44,46]
Australia	2	[25,27]
The Netherlands	2	[21,24]

Abbreviations: GC-MS, gas chromatography–mass spectrometry; HPLC-ECD, high-performance liquid chromatography with electrochemical detection; LC-MS, liquid chromatography–mass spectrometry; UPLC-MS/MS, ultra-performance liquid chromatography–tandem mass spectrometry).

Sample sizes varied widely, ranging from small metabolic studies involving <10 participants to large observational cohorts, including studies with nearly 500 participants [21]. Studies were predominantly conducted in the United States, Europe, and Japan. Study populations encompassed healthy adults, individuals with cigarette smoking exposure, and participants with specific disease states, including metabolic syndrome, obesity, diabetes, inherited lipid disorders, and prostate cancer.

### Methodological variability in urinary α-CEHC measurement

Substantial methodological heterogeneity was observed across studies in urine collection protocols, analytical techniques, and reporting and normalization strategies for urinary α-CEHC. These differences contribute to the variability in reported concentrations and limit direct comparison across studies (Table 1). Most studies (18 of 34) used 24-h urine collections [17,18,[21], [22], [23], [24],[26], [27], [28],[31], [32], [33],[40], [41], [42], [43],^46^,^50^], whereas others employed single spot urine samples [25,29,38,44,45,47,49,51], serial pooled urine collections for a combined sample to equal a 24-h urine collection [6,34,36,37,39], or both spot sample and 24-h collections [35,48]. One study reported a single unspecified time point that was categorized as not applicable [49]. Reporting of fasting status and timing of urine collection relative to vitamin E dosing or supplementation varied across studies and was not consistently specified.

Analytical approaches used to quantify urinary α-CEHC varied across studies and over time and included: GC-MS, high-performance liquid chromatography with electrochemical detection (HPLC-ECD), liquid chromatography–mass spectrometry (LC-MS), and ultra-performance liquid chromatography–tandem mass spectrometry (UPLC-MS/MS) (Table 1). In general, studies prior to 2008 primarily employed GC-MS or HPLC-ECD, whereas later studies more frequently employed LC-MS and UPLC-MS/MS–based methods. The more recent studies used more sensitive technologies. Across studies using MS-based methods, the reported urinary α-CEHC concentrations fell within similar ranges between studies (Supplementary Data 1). However, reporting of conjugate hydrolysis procedures and limits of detection was inconsistent. Conjugate hydrolysis techniques included enzymatic (glucuronidase or both glucuronidase/sulfatase) and acid (HCl incubation at 60°C), and some used both enzyme and acid. A few studies used metabolomics approaches (e.g., time of flight MS) with custom synthesized conjugated catabolites (such as α-CEHC-glycine, α-CEHC glycine-glucuronide, and α-CEHC-taurine) to evaluate types of conjugates excreted [21,40].

Additionally, creatinine normalization was the most commonly used reporting approach. Among the 19 studies reporting creatinine-normalized values, baseline urinary α-CEHC concentrations clustered within a relatively narrow range across populations and study designs [6,[21], [22], [23], [24], [25], [26], [27], [28],^30^,[32], [33], [34], [35], [36],^40^,^45^,^47^,^49^]. For consistency, we standardized creatinine-normalized values to μmol/mol creatinine (Table 2 and Supplementary Data 1). In studies of participants not receiving vitamin E supplementation, or at baseline prior to supplementation, urinary α-CEHC concentrations ranged from ∼12 to 620 μmol/mol creatinine (Supplementary Data 1). Furthermore, 7 studies reported total daily urinary α-CEHC excretion, 4 studies reported α-CEHC concentrations per liter of urine without adjustment for urine volume, and 4 studies expressed results as a percentage of baseline or cumulative excretion without reporting absolute concentrations (Table 1). One study reported creatinine standardization, but α-CEHC concentrations were reported as percentages of baseline values and was therefore classified as “other.” In general, studies reporting total daily excretion showed values within expected adult urinary output ranges as compared to creatinine-normalized measurements (Table 2 and Supplementary Data 1).

**TABLE 2:** Vitamin supplement size in relationship to α-CEHC increase

Reference	Population	Vitamin E supplement^1^	Daily dose	Length of intervention	Hydrolysis technique	Analysis technique	Urinary α-CEHC mean ± SEM, μmol/mol creatinine (original units)		
							Baseline	Postsupplement	Mean increment
Beaver 2025 [26]	Adults with ≥3 MetS criteria *n* = 38, 17% female	*RRR*-α-T (food-derived)	10.3 mg α-T per 58 g almonds	12 wk	Acidified with HCl, 1 h 60°C	UPLC-MS	71 ± 53 (0.63 ± 0.09 μmol/g)	89 ± 61 (0.78 ± 0.10 μmol/g)	18
Bruno 2005 [32]	Healthy adults (smokers, 18–35 y) *n* = 10, 40% female	d_3_-*RRR*-α-TAc and d_6_-*all-rac*-α-TAc	75 mg each (150 mg/d total)	6 d	Enzymatic hydrolysis: glucuronidase	GC-MS	63 ± 17 (560 ± 150 nmol/g)^2^	249 ± 34 (2200 ± 300 nmol/g)^2^	186
[32]	Healthy adults (nonsmokers, 18–35 y) *n* = 10, 40% female	d_3_-*RRR*-α-Tac and d_6_-*all-rac*-α-TAc	75 mg each (150 mg/day total)	6 d	Enzymatic hydrolysis: glucuronidase	GC-MS	61 ± 23 (540 ± 200 nmol/g)^2^	328 ± 101 (2905 ± 900 nmol/g)^2^	267
Devaraj 2008 [28]	Adults with ≥3 MetS criteria (56 ± 11 y) *n* = 20, 70% female	Placebo	0 mg	6 wk	Enzymatic hydrolysis: glucuronidase	LC-MS	339 ± 113 (3 ± 1 μmol/g)^2^	226 ± 113 (2 ± 1 μmol/g)^2^	0
[28]	Adults with ≥3 MetS criteria (50 ± 9 y) *n* = 20, 90% female	γ-T	Unknown α-T	6 wk	Enzymatic hydrolysis: glucuronidase	LC-MS	113 ± 113 (1 ± 1 μmol/g)^2^	226 ± 113 (2 ± 1 μmol/g)^2^	113
[28]	Adults with ≥3 MetS criteria (51 ± 11 y) *n* = 20, 80% female	*RRR*-α-T	800 mg α-T	6 wk	Enzymatic hydrolysis: glucuronidase	LC-MS	226 ± 113 (2 ± 1 μmol/g)^2^	2034 ± 339 (18 ± 3 μmol/g)^2^	1808
[28]	Adults with ≥3 MetS criteria (57 ± 14 y) *n* = 20, 65% female	γ-T + *RRR*-α-T	800 mg α-T^1^	6 wk	Enzymatic hydrolysis: glucuronidase	LC-MS	283 ± 113 (2.5 ± 1 μmol/g)^2^	904 ± 113 (18 ± 1 μmol/g)^2^	621
Imai 2011 [46]	Healthy college students (20–33 y) *n* = 76, 100% female	0, diet only 13 μmol/d (5.6 mg/d)	0 mg/d	Diet only	Enzymatic hydrolysis: glucuronidase	HPLC-ECD	50 ± 33 (0.444 ± 0.292 μmol/d)^3^	—	0
[46]	Healthy college students (20 ± 2 y) *n* = 10, 100% male	α-TAc	21 μmol/d (9 mg)	4 d	Enzymatic hydrolysis: glucuronidase	HPLC-ECD	84 ± 22 (0.74 ± 0.2 μmol/d)^3^	106 ± 22	22
[46]	—	α-TAc	63 μmol/d (27 mg)	4 d	Enzymatic hydrolysis: glucuronidase	HPLC-ECD	84 ± 22 (0.74 ± 0.2 μmol/d)^3^	214 ±113	130
[46]	—	α-TAc	125 μmol/d (54 mg)	4 d	Enzymatic hydrolysis: glucuronidase	HPLC-ECD	84 ± 22 (0.74 ± 0.2 μmol/d)^3^	377 ± 113	293
Clarke et al. [27] 2006	Adults with type II diabetes (64 ± 7 y) *n* = 18, 28% female	*RRR*-α-T	500 mg	3 wk	Enzymatic hydrolysis: glucuronidase	GC-MS	210 ± 40	7840 ± 140	7630
[27]	Adults with type II diabetes (58 ± 5 y) *n* = 18, 37% female	Mixed Ts	75 mg α-T	3 wk	Enzymatic hydrolysis: glucuronidase	GC-MS	220 ± 30	1290 ± 210	1070
[27]	Adults with type II diabetes (64 ± 7 y) *n* = 18, 11% female	Placebo	0 mg	3 wk	Enzymatic hydrolysis: glucuronidase	GC-MS	190 ± 60	170 ± 30	0
Michels et al. [45] 2018	Healthy older adults (63 ± 6 y) *n* = 32, 68% female	*RRR*-α-T (food-derived)	7.7–10.4 mg α-T per 57 g hazelnuts	16 wk	Acidified with HCl, 1 h 60°C	UPLC-MS	95 ± 9 (0.84 ± 0.08 μmol/g)	127 ± 10 (1.12 ± 0.09 μmol/g)	32
Morinobu et al. [44] 2003	Healthy adults (29± 3 y) *n* = 14, 100% male	*RRR*-α-T	800 mg/d	14 d	Enzymatic hydrolysis: glucuronidase	HPLC-ECD	270 ± 15	8010 ± 40	7740
[44]	—	*RRR*-α-T	800 mg/d	28 d	Enzymatic hydrolysis: glucuronidase	HPLC-ECD	270 ± 15	9760 ± 492	9490
Traber et al. [18] 1998	Healthy adults (27–52 y) *n* = 6, 83% female	d_3_-*RRR*-α-TAc + d_6_-*all-rac*-α-TAc	150 mg each (300 mg total)	Single dose	Enzymatic hydrolysis: glucuronidase/sulfatase	GC-MS (multiday preparation methods)	113 ± 22 (1.0 ± 0.2 μmol/d)^3^	421 ± 93 (3.7 ± 0.8 μmol/d)^3^	308
Traber et al. [6] 2021	Healthy adults (18–40 y) *n* = 10, 100% female	*RRR*-α-T	60 mg deuterated α-T	Once	Acidified with HCl, 1 h 60°C	UPLC-MS	135 ± 34 (1.2 ± 0.3 μmol/g)	655 ± 135 (5.8 ± 1.2 μmol/g)	520
Yoshikawa et al. [43] 2005	Healthy adults (28 ± 3 y) γ-tocopherol group) *n* = 7, 100% male	2 x (Capsule = deuterated α-T 2.5 mg; deuterated β-T 1.4 mg; deuterated γ-T 93.2 mg; deuterated T-δ: 2.3 mg)	5 mg α-T	4 wk	Enzymatic hydrolysis: glucuronidase	HPLC-ECD	84 ± 22 (0.8 ± 0.2 μmol/d)^2^^,^^3^	124 ± 22 (1.1 ± 0.2 μmol/d)^2^^,^^3^	29
[43]	Healthy adults (28 ± 3 y) (α-tocopherol group) *n* = 6, 100% male	α-T	5 mg α-T	4 wk	Enzymatic hydrolysis: glucuronidase	HPLC-ECD	79 ± 11 (0. ± 0.1 μmol/d)^2^^,^^3^	79 ± 11 (0. ± 0.1 μmol/d)^2^^,^^3^	0

Abbreviations: α-CEHC, α-carboxyethyl hydroxychromanol; α-T, α-tocopherol; α-TAc, α-tocopheryl acetate; β-T, β-tocopherol; γ-T, γ-tocopherol; GC-MS, gas chromatography–mass spectrometry; HCl, hydrochloric acid; HPLC-ECD, high-performance liquid chromatography with electrochemical detection; LC-MS, liquid chromatography–mass spectrometry; MetS, metabolic syndrome; SEM, standard error of the mean; Ts, tocopherols; T-δ, δ-tocopherol; UPLC-MS/MS, ultra-performance liquid chromatography–tandem mass spectrometry.

1 XXXXX.

2 Data estimated from graph shown in reference cited.

3 In micromoles per day, assuming 1 g creatinine excreted per day.

For standardization and convenience purposes, a customary practice is to estimate 24-h urinary excretion rates of protein, sodium, potassium, calcium, magnesium, urea, and uric acid, which are generally calculated under the assumption of a daily creatinine excretion of 1 g, with a urinary creatinine concentration of 100 mg/dL corresponding to a 24-h urine volume of 1 L [52]. Although this approach is inappropriate in settings of changing serum creatinine and may yield inaccurate estimates at extremes of age or body size, it was applied here to facilitate approximate cross-study comparability [52]. Accordingly, for studies reporting daily excretion values, the assumption made was that 1 g creatinine is excreted daily and was used to convert 24-h results to creatinine-normalized measures. Studies reporting α-CEHC concentrations in micromoles per liter without volume correction were recorded as reported, limiting cross-study comparability for these studies.

Finally, substantial heterogeneity was observed in urine collection protocols across included studies. Of the studies reporting collection methods, 18 employed 24-h urine collections, 8 relied on single time point (spot) urine samples, 2 studies reported a combination of 24-h urine and spot sample collections, 5 studies reported serial pooled collections, and 1 study reported a single unspecified time point that was categorized as “not applicable” (Table 1).

As mentioned, 5 studies collected urine in discrete intervals rather than as a single pooled 24-h sample [6,34,36,37,39]. For example, in 2 controlled supplementation tracer studies [6,36], urinary α-CEHC was measured across 3 consecutive 8-h collection periods spanning 0–24 h during each intervention phase, with sampling repeated across multiple days. In these studies, α-CEHC concentrations measured across intervals were comparable to those reported in other intervention-based studies employing full 24-h collections.

### Responsiveness of urinary α-CEHC to vitamin E intake or supplementation

**FIGURE 3:**
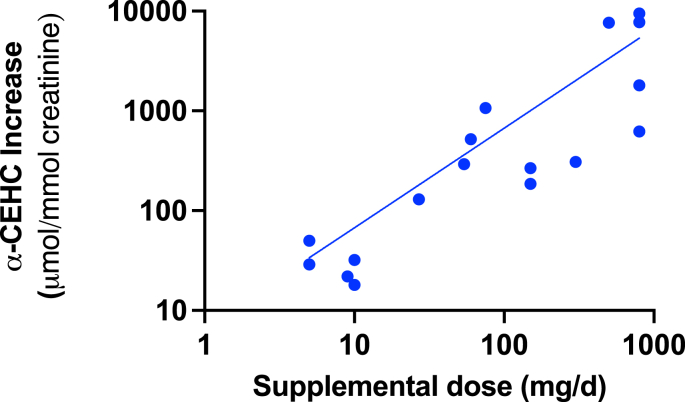
Relationship between supplemental α-tocopherol dose and urinary α-carboxyethyl hydroxychromanol (α-CEHC). The increases in urinary α-CEHC excretion in response to supplemental dose (in milligrams per day) shown in Table 2 (for all nonzero values) was plotted on a log-log scale. Pearson’s correlation calculations resulted in an *R*^2^ = 0.7819 and a 2-tailed *P* < 0.0001.

Across the intervention, feeding, and tracer studies, urinary α-CEHC increased in response to vitamin E intake. Ten supplementation studies in which both baseline and postsupplementation urinary α-CEHC values, reported either as creatinine-normalized concentrations or as total daily excretion [6,18,[26], [27], [28],^32^,[43], [44], [45], [46]], were evaluated to assess the relationship between these 2 parameters. Baseline urinary α-CEHC values in these studies ranged from ∼50 to 339 μmol/mol creatinine, whereas final mean postsupplementation values ranged from ∼89 to 9760 μmol/mol creatinine (Table 2). The increment in urinary α-CEHC excretion reported was correlated with the dose of the α-tocopherol supplement (*P* < 0.0001, Figure 3). Although lengths of dosing intervals are shown in Table 2, no corrections were made for how long the vitamin E supplement was administered. Largely, changes appeared to occur in the first day or so of supplementation as measured with labeled α-tocopherol, suggesting urinary α-CEHC excretion occurs fairly rapidly with changes in vitamin E intake. Early metabolic and tracer studies captured rapid changes in urinary α-CEHC after α-tocopherol exposure. For example, a controlled supplementation tracer study reported measurable increases in urinary α-CEHC within 24–72 h after α-tocopherol administration, including isotopically labeled forms, with increases observed shortly after dosing and detectable within the first days of exposure [18].

Longer supplementation and feeding studies reported sustained elevations in urinary α-CEHC over periods ranging from several days to weeks. For example, a nonrandomized experimental study employing 28 days of tocopherol supplementation (1200 IU/d; 800 mg) reported elevated excretion persisting across the intervention period [44]. Food-based dietary interventions also reported increases in urinary α-CEHC, generally of smaller magnitude than high-dose supplementation. For example, one study reported an increase in creatinine-normalized urinary α-CEHC after 16 wk of hazelnut consumption (∼57 g/d) in healthy older adults, corresponding to an increase of ∼8.8 mg/d in dietary α-tocopherol intake [45]. Similarly, another study reported increased urinary α-CEHC excretion in adults with metabolic syndrome after 12 wk of almond consumption (58 g/d; 10.3 mg α-tocopherol) [26].

The magnitude of urinary α-CEHC response varied across studies by form and dose of vitamin E administered (Table 2). Studies administering synthetic α-tocopherol forms, isotopically labeled tracers, or high-dose α-tocopherol supplements reported higher absolute urinary α-CEHC values over shorter time frames compared with dietary interventions providing lower daily intakes [26,45]. Although normalization strategy and reporting units differed across studies, all studies described increases in urinary α-CEHC with higher vitamin E intake, and extraordinarily huge increases were reported after prolonged intakes of high-dose α-tocopherol supplements [[27], [44]].

### Metabolic syndrome

Studies including individuals with metabolic syndrome reported lower urinary α-CEHC excretion and differences in response to α-tocopherol intake compared with metabolically healthy populations. In a controlled tracer study, a single 15-mg oral dose of d_6_-*RRR*-α-tocopherol was administered in 1 of 4 milk-based beverages to adults with metabolic syndrome and metabolically healthy controls [36]. Despite receiving the same α-tocopherol dose, individuals with metabolic syndrome exhibited lower urinary α-CEHC excretion [36]. This pattern was observed in both males and females when comparisons were made within sex (healthy males compared with males with metabolic syndrome; healthy females compared with females with metabolic syndrome) [36]. Another study evaluated adults with metabolic syndrome receiving 800 mg/d of *RRR*-α-tocopherol for 6 wk, administered either alone or in combination with γ-tocopherol [28]. In this study, urinary α-CEHC increased significantly from baseline after supplementation in both intervention groups (*P* < 0.001). However, postsupplementation urinary α-CEHC excretion was significantly greater in participants receiving α-tocopherol alone compared with those receiving the combined α- and γ-tocopherol intervention (*P* < 0.001) [28]. The impact of supplementation with other tocochromanols on α-tocopherol catabolism and α-CEHC excretion is unclear.

A food-based randomized controlled trial reported that almond consumption by adults with metabolic syndrome was associated with an estimated 18 μmol/mol creatinine increase in α-tocopherol intake of ∼10 mg/d [26]. Additionally, almond intake was accompanied by increases in urinary α-CEHC, along with changes in plasma vitamin E indices, including an increase in the plasma α-tocopherol/cholesterol ratio and a decrease in the γ-tocopherol/cholesterol ratio [26]. In contrast, studies conducted in metabolically healthy populations reported larger increases in urinary α-CEHC after relatively similar increases in dietary vitamin E intake. For example, healthy adults aged ≥55 y with low habitual dietary vitamin E intake who consumed an additional ∼57 g/d of dry-roasted hazelnuts for 16 wk—corresponding to an estimated increase in α-tocopherol intake of 7.7–10.4 mg/d—exhibited a 33% increase in creatinine-normalized urinary α-CEHC (32 μmol/mol creatinine; or as published, from 0.84 to 1.14 μmol/g creatinine; *P* = 0.0006) [45].

### Biomarker of oxidized α-tocopherol

An analytical validation study of individuals with type I diabetes and matched healthy controls under habitual dietary conditions reported that the children with diabetes had >10-fold increased excretion of an oxidized catabolite, α-tocopheronolactone, as well as urinary α-CEHC values double those of control children [49]. The authors posited that the oxidative stress in the children with diabetes was causing α-tocopherol oxidation [49].

In a cross-sectional study, urine samples collected from the population-based Netherlands Epidemiology of Obesity (NEO) Study quantified both α-CEHC and α-tocopheronic acid hydroquinone (α-TLHQ; catabolite of tocopheryl quinone, Figure 1) [21]. Remarkably, the α-CEHC-GLU (glucuronide conjugate) was similar to α-CEHC reported in other studies [91.0 (range, 62.3–139.4) nmol/mmol creatinine], but α-TLHQ-GLU [1822.5 (range, 1339.3–2744.8) nmol/mmol creatinine] was elevated about 20 times that of α-CEHC [21]. These authors also reported associations of vitamin E catabolites with other catabolites, as well as with obesity and lifestyle factors [[53], [54], [55]]. Further studies are needed to evaluate the utility of α-TLHQ as a biomarker of oxidized α-tocopherol.

### Additional clinical contexts

Several studies evaluated urinary α-CEHC excretion in specific clinical or exposure contexts beyond metabolically healthy populations and metabolic syndrome. In a short-term randomized controlled trial, one study reported marked increases in urinary γ-CEHC and δ-CEHC after γ-tocopherol-rich supplementation in males with localized prostate cancer [29]. However, in this study, urinary α-CEHC concentrations were reported as volume-normalized values (micromoles per liter) rather than creatinine-normalized or total excretion measures, limiting direct comparison with other studies included in this review [29].

One cross-sectional study evaluated urinary α-CEHC excretion in participants with familial hypobetalipoproteinemia [25]. Adults with heterozygous familial hypobetalipoproteinemia exhibited urinary α-CEHC excretion within ranges reported for healthy populations, despite low circulating tocopherol concentrations [25]. In the same study, urinary α-CEHC excretion was reported in 2 children with homozygous familial hypobetalipoproteinemia after vitamin E supplementation, although no comparison to healthy pediatric controls was provided [25].

Several studies examined urinary α-CEHC excretion in smokers. One tracer study reported lower postsupplementation urinary α-CEHC excretion in smokers compared with matched nonsmokers following 6 d of deuterium-labeled α-tocopherol supplementation, despite similar baseline urinary α-CEHC values [56]. Similarly, an observational cross-sectional study reported lower 24-h urinary α-CEHC excretion in smokers compared with matched nonsmokers under habitual dietary conditions [22]. A randomized controlled trial reported creatinine-normalized urinary α-CEHC values during a short-term smoking cessation intervention; however, variability attributed to internal standard performance was noted, and changes in urinary α-CEHC over time were reported as variable [30].

## Discussion

This scoping review systematically mapped >2 decades of human research evaluating urinary α-CEHC in relation to vitamin E intake, catabolism, and physiological context. Across diverse study designs, populations, and intervention types, urinary α-CEHC consistently increased after vitamin E exposure, indicating a reproducible response to intake and metabolic processing. However, substantial methodological heterogeneity across studies limited direct comparability of reported values. These findings indicate that urinary α-CEHC is a responsive measure of supplemental vitamin E intake and catabolism (Figure 3), but that interpretation is strongly influenced by study context and methodology.

### Responsiveness of urinary α-CEHC to vitamin E intake

A central finding of this review is the consistent increase in urinary α-CEHC after vitamin E intake across both short-term and sustained interventions. Early tracer and metabolic studies demonstrated that α-CEHC appears in urine within 24–72 h of α-tocopherol administration, including after isotopically labeled dosing [31,35,48]. These observations indicate that urinary α-CEHC reflects recent intake and downstream metabolic processing rather than longer-term tissue accumulation.

Sustained elevations in urinary α-CEHC were also reported in longer supplementation and feeding studies, including both pharmacologic synthetic dosing and food-based interventions (Table 2). Notably, increases in urinary α-CEHC were observed not only with high-dose supplementation but also after modest increases in dietary α-tocopherol intake from whole foods such as almonds and hazelnuts [26,45]. Across both high-dose supplementation and food-based intervention studies, urinary α-CEHC increased in a manner that was directionally consistent despite variation in dose, duration, and intervention type, supporting its responsiveness across a range of exposure levels (Table 2, Figure 3). It is important to note that basal dietary intake was variably controlled across supplementation studies. In most of these studies, efforts were made to account for background dietary intake, where participants were instructed to avoid foods, beverages, and supplements containing vitamin E during the study period. Other studies collected dietary records during investigation to monitor for potential differences in nutrient intake that could influence vitamin E intake. However, the extent and duration of dietary standardization varied across supplementation studies. As a result, variability in background vitamin E intake may contribute to differences in baseline urinary α-CEHC excretion and therefore complicate interpretation of the magnitude of responses observed following supplementation.

Studies that administered synthetic, isotopically labeled, or high-dose α-tocopherol generally reported higher absolute urinary α-CEHC values over shorter time frames than did studies with smaller doses, but sensitivity of the methodology to changes may be one explanation as to why high doses or labeled α-tocopherol were used. It may be that food-based interventions produce smaller increases but that with the newer methodologies, these changes are detectable. For example, increases in response to almonds were observed by 4 wk [26]. Additionally, d_6_-α-CEHC could be detected in the 0–8-h urine collections after a 15 mg dose of d_6_-α-tocopherol [36]. These patterns are consistent with urinary α-CEHC reflecting the metabolic handling of α-tocopherol once intake exceeds immediate physiological requirements.

### Clinical and physiological contexts

Beyond responsiveness to intake, urinary α-CEHC varied across physiological and clinical contexts. One of the most provocative patterns identified was reduced urinary α-CEHC excretion in individuals with metabolic syndrome compared with metabolically healthy controls despite comparable or higher vitamin E exposure [36]. These findings suggest that urinary α-CEHC reflects differences in vitamin E handling across metabolic states and that considering physiological context is important when interpreting urinary excretion patterns. Additionally, persons with metabolic syndrome may have higher vitamin E requirements, but further studies are needed to confirm this observation.

Additional clinical contexts further illustrated both the utility and limitations of urinary α-CEHC. In inherited lipid disorders, normal urinary α-CEHC excretion in the presence of low circulating α-tocopherol concentrations suggests preserved metabolic conversion despite altered transport [25]. These results reflect the complementary information provided by urinary catabolites but also suggest that more studies are needed to determine expected urinary α-CEHC excretion in metabolic transport disorders in response to vitamin E supplementation. Such findings will have important implications in disorders of altered lipoprotein transport, such as ABL, where high-dose vitamin E supplementation is the primary treatment and validating the response to supplementation is essential.

In smokers, consistently lower urinary α-CEHC excretion compared with nonsmokers, despite similar baseline values or controlled supplementation, aligns with observations of altered antioxidant dynamics in the context of smoking exposure [22,32,[56], [57], [58]].

Collectively, these findings indicate that urinary α-CEHC reflects not only α-tocopherol intake but also underlying metabolic and physiological conditions. Most importantly, having comparisons between healthy urinary α-CEHC levels and those of individuals affected by different metabolic processing disorders can guide treatment.

### Methodological variability and its implications

Despite consistent biological responsiveness, there was substantive methodological variability of the urinary α-CEHC literature. Differences in urine collection protocols, normalization strategies, analytical platforms, and reporting practices limited wider cross-study comparison of absolute values and complicated interpretation of between-study variability.

An additional source of variability can be attributed to differences in the stereochemical forms of α-tocopherol used across studies. Although natural vitamin E (*RRR*-α-tocopherol) contains exclusively 2*R*-α-tocopherol stereoisomers, synthetic preparations such as *all-rac*-α-tocopherol include an equimolar mixture of 8 stereoisomers containing both 2*R-* and 2*S-*α-tocopherol configurations [59]. The hepatic α-tocopherol transfer protein (α-TTP) preferentially binds and retains 2*R*-stereoisomers, whereas 2*S-*α-tocopherol stereoisomers are instead rapidly metabolized in the liver via cytochrome P450-initiatied ω-hydroxylation followed by β-oxidation and converted into α-CEHC metabolites [18]. As a result, supplementation with synthetic α-tocopherol may result in higher urinary α-CEHC measurements as compared to natural vitamin E forms at equivalent doses. These differences in stereochemical composition of vitamin E forms should be considered when comparing urinary α-CEHC across studies.

Creatinine normalization was the most frequently employed reporting approach and was associated with tighter clustering of baseline urinary α-CEHC values across populations and study designs. In contrast, studies reporting α-CEHC concentrations per liter of urine without adjustment for dilution could not be readily compared with creatinine-normalized studies or those reporting total excretion within a specified time, most commonly 24 h. To facilitate approximate cross-study comparison in the presence of this heterogeneity, standardized assumptions regarding daily creatinine excretion were applied. Although such approximations may introduce imprecision—particularly at extremes of age, body size, or renal function—they were used solely to enable relative comparison across studies and are unlikely to alter the observed qualitative patterns of α-CEHC responsiveness. However, it is still important to recognize that creatinine-adjusted urinary α-CEHC values may introduce variability between individuals with differing muscle mass or in spot urine samples collected at different time points throughout the day due to diurnal variation. Therefore, these factors should be considered when interpreting creatine-normalized α-CEHC measurements and when comparing results across studies employing different normalization and urine collection protocols. For example, adults with metabolic syndrome exhibited greater 24-h urinary creatinine excretion than healthy participants (1.96 ± 0.10 g compared with 1.53 ± 0.10 g, respectively; *P* = 0.008) [36].

Additionally, variation in urine collection timing has important implications for the interpretation of urinary α-CEHC as a biomarker of vitamin E status. In particular, reliance on single time point (spot) urine samples raises questions regarding temporal variability and whether casual collections adequately reflect daily α-tocopherol catabolism. Evidence from intervention studies incorporating segmented urine collections provides partial reassurance in this regard. Studies that measured urinary α-CEHC across multiple intervals within a 24 h period demonstrated relatively stable excretion patterns over time, and pooled or interval-based values were broadly consistent with those obtained from complete 24-h collections [6,36]. These findings suggest that urinary α-CEHC excretion may be sufficiently integrated over time to permit estimation of noncontinuous sampling, although more studies combining and comparing this approach with nonserial 24-h collections are needed to validate this approach.

Overall, there are limitations in the number of studies reporting data directly comparing spot, interval-based, and full 24-h collections. Further, reporting practices are inconsistent. As a result, although spot urine samples may offer a practical proxy for α-tocopherol exposure in some contexts, particularly in large-scale or population-based studies, further work is needed to define the conditions under which spot collections can reliably approximate 24-h excretion and to characterize within-day variability across diverse populations.

### Recommendations for estimating urinary α-CEHC in adequately nourished populations

Based on the evidence synthesized in this review, several conditions appear necessary to obtain a meaningful estimate of urinary α-CEHC in adequately nourished populations. First, population context must be carefully defined. Even among individuals without overt vitamin E deficiency, urinary α-CEHC excretion varies with metabolic status, smoking exposure, and inherited lipid disorders, indicating that “adequate α-tocopherol intake” does not imply uniform metabolic processing. Consequently, interpretation of urinary α-CEHC in ostensibly healthy cohorts should account for metabolic phenotype rather than relying solely on dietary intake or circulating tocopherol concentrations. Especially important may be 2 common single nucleotide polymorphisms (SNPs) in the *CYP4F2* gene causing significant alterations in tocochromanol ω-hydroxylase activity [15]. The W12G variant (rs3093105) showed increased enzyme activity toward all tocochromanols, whereas the V433M variant (rs2108622) showed reduced enzyme activity toward tocopherols but not tocotrienols [15]. The effect of these SNPs on vitamin E status and the response to vitamin E supplementation in humans is relatively unstudied [60,61].

Second, urine collection and normalization strategies are central to interpretability. Complete 24-h urine collections provide the most direct estimate of daily α-CEHC excretion and remain the reference standard where feasible. However, evidence from studies employing pooled serial collections suggests that α-CEHC excretion may be sufficiently integrated over time to permit estimation of daily excretion, although more studies are needed to validate this method.

Finally, creatinine normalization appears to be the most consistently applied approach for estimating urinary α-CEHC across adequately nourished populations, yielding relatively narrow baseline ranges across diverse study designs. When total daily excretion is reported instead of creatinine standardization, documentation of collection completeness and timing is essential to enable comparison with creatinine-normalized results. In contrast, volume-normalized reporting without adjustment for dilution appears unreliable and should be avoided.

Together, these considerations define a minimal framework under which urinary α-CEHC can be interpreted as an integrated marker of α-tocopherol intake and catabolism in populations with adequate intakes.

### Implications for biomarker development and future research

Taken together, the findings of this scoping review indicate that urinary α-CEHC is a responsive marker of vitamin E metabolic handling whose interpretation is highly dependent on physiological context and methodological approach. Although its consistent response to α-tocopherol intake supports continued investigation, methodological standardization is necessary before urinary α-CEHC can be meaningfully compared across studies or considered for broader clinical application. An additional consideration in interpreting urinary α-CEHC as a biomarker of α-tocopherol intake is that α-CEHC metabolites can also be generated from α-tocotrienol. Although dietary α-tocotrienol intake is generally low in Western populations, it may be more substantial in regions where palm oil and other tocotrienol-rich oils are commonly consumed. Therefore, it is possible that urinary α-CEHC is not specific to α-tocopherol metabolism in global populations with higher tocotrienol exposure. Future studies should include total tocochromanol intake when interpreting CEHC measurements, especially in regions where tocotrienol-rich oils are more appreciably consumed. For example, the reported daily total tocotrienol intake in Poland was ∼2 mg [62], similar to values reported for Japan, where α-tocopherol intakes were 8–10 mg [63].

Another important consideration when interpreting urinary α-CEHC as a biomarker of α-tocopherol intake is the relatively small proportion of α-CEHC eliminated (estimated at 1%–2% [6]) relative to α-tocopherol intake. The small proportion of oral α-tocopherol that is excreted as α-CEHC metabolites reflects the tightly regulated handling and its preferential retention in the body mediated by the hepatic α-TTP. Therefore, rather than urinary α-CEHC measurements serving as a biomarker for total vitamin E intake, they should be interpreted primarily as a marker of α-tocopherol daily intake in excess of needs, as was suggested by the sharper increase in vitamin E metabolite excretion that occurred with an intake of ∼9 mg α-tocopherol/d [23]. Further, the consistent increases in urinary α-CEHC after supplementation across multiple study designs supports its measurement as a sensitive indicator of increased α-tocopherol exposure, which will have especially important implications for disorders in which vitamin E supplementation represents the primary treatment and monitoring response to therapy remains challenging, such as ABL [4].

Although the available evidence from this review supports urinary α-CEHC as a responsive marker of vitamin E catabolism, interpretation of this biomarker remains influenced by several biological, methodological, and analytical sources of variability. As summarized in Table 3, heterogeneity in urine collection protocols, normalization approaches, and background dietary vitamin E intake may contribute to differences in baseline α-CEHC excretion and limit comparability across studies. In addition, biological factors—including differences in α-tocopherol stereoisomers, potential contributions from other tocochromanols such as α-tocotrienol, and the relatively small proportion of ingested α-tocopherol recovered as CEHC—complicate interpretation of urinary α-CEHC as a direct measure of vitamin E intake. Finally, variability in analytical platforms and the sensitivity of different chromatographic approaches further highlights the need for methodological harmonization. Therefore, we suggest future research aim to refine the interpretation urinary α-CEHC as a biomarker of vitamin E catabolic flux by addressing these limitations through standardized urine collection and normalization protocols, controlled supplementation studies, stereoisomer-specific investigations, and interlaboratory analytical validation (Table 3). Establishing reference ranges across physiological states and evaluating urinary α-CEHC alongside complementary vitamin E catabolites may further refine interpretation. Studies in populations with altered lipid transport, metabolic dysfunction, or increased oxidative demand will be particularly informative for clarifying the contexts in which urinary α-CEHC provides added value beyond circulating tocopherol measurements. This clarification will have immense benefit for disorders in which high-dose vitamin E supplementation is the primary treatment––such as ataxia with vitamin E deficiency and ABL––as there is currently no reliable approach to validating response to vitamin E supplementation in these patients [4].

**TABLE 3:** Current limitations of urinary α-CEHC as a biomarker of vitamin E status and directions for future research

Limitation	Description	Implications for biomarker interpretation	Future research directions
Urine collection variability	Studies used spot urine, pooled collections, or 24-h urine samples	Limits comparability across studies	Standardized urine collection protocols (preferably 24-h collections); more studies comparing pooled serial collections as an estimation of daily excretion to validate this method
Suitability of urine-based assessments over plasma measurements	Urine-based assessments may not be well suited for use outside research settings	The use of plasma vs. urine is likely to favor urine because this is technically easier and is a collection of the material excreted from the body	More studies comparing plasma and urine α-CEHC
Normalization methods	Creatinine normalization vs. total volume studies and limitations of creatinine-adjusted normalization	Differences in normalization methods limits comparability across studies; creatinine-adjusted normalization influenced by muscle mass and diurnal variation	Evaluate optimal normalization strategies and standardize normalization protocols (preferably creatinine-adjusted normalization); future studies exploring the influence of muscle mass and diurnal variation in creatinine normalization on urinary α-CEHC excretion
Dietary background variability	Basal vitamin E intake inconsistently controlled	Background diet may influence baseline α-CEHC excretion	Controlled supplementation studies with standardized protocols for controlling basal vitamin E intake and thorough dietary record documentation prior to and during investigations
Vitamin E form variability	Natural vs. synthetic α-tocopherol; 2R vs. 2S stereoisomers	Different catabolic rates may influence CEHC production	Standardized reporting of vitamin E forms; more studies comparing excretion between stereoisomers at nominal equivalent doses
Biomarker specificity	α-CEHC may also derive from α-tocotrienol metabolism	May affect specificity in populations with higher tocotrienol intake	Future studies should include total tocochromanol intake when interpreting CEHC measurements; studies comparing α-CEHC excretion between individuals and populations with different tocotrienol intake levels (from natural food sources)
Fraction of oral intake recovered as CEHC	Only a small proportion of α-tocopherol intake is excreted as CEHC	CEHC reflects catabolic flux rather than total intake	More quantitative controlled supplementation tracer investigations to evaluate the regulation of the production of α-CEHC
Analytical variability	Different LC-MS and chromatographic approaches	Limits cross-study comparability	Interlaboratory method harmonization; studies comparing sensitivity between chromatographic approaches

Abbreviations: CEHC, carboxyethyl hydroxychromanol; LC-MS, liquid chromatography–mass spectrometry.

## Author contributions

The authors’ responsibilities were as follows – YB designed the study, consulted librarians and conducted the search, and drafted the initial manuscript; YB and MGT reviewed and selected studies, extracted data from articles, generated study figures, and reviewed and commented on subsequent drafts of the manuscript; YB and MGT had primary responsibility for final content; and all authors: read and approved the final version of the manuscript.

## Declaration of generative AI and AI-assisted technologies in the writing process

The authors declare that no generative AI or AI-assisted technologies were used in the writing of this manuscript.

## Funding

The authors reported no funding received for this study.

## Conflicts of interest

Maret Traber is an Editorial Board Member for the Journal of Nutrition and played no role in the Journal’s evaluation of the manuscript. The other authors reports no conflicts of interest.
