# Supplementary material for: Urinary α-Carboxyethyl Hydroxychromanol (α-CEHC)—A Biomarker of Vitamin E Intake: A Scoping Review
Source: J Nutr. 2026 Mar 21;156(5):101486. doi: 10.1016/j.tjnut.2026.101486 (PMC13197953; doi:10.1016/j.tjnut.2026.101486)
Supplement: Multimedia component 1 [file mmc1.pdf]

Search Strategy: Urinary α-Carboxyethyl Hydroxychromanol (α-CEHC) as a Biomarker of Vitamin E Intake: A Scoping Review

Corresponding Author:
Yochana Benchetrit, BS
Temerty Faculty of Medicine, University of Toronto
Email: yochana.benchetrit@mail.utoronto.ca
Telephone: 647-972-9966
417 Sackville Street, Toronto, Canada, M4X1S8

Other notes on search strategy construction:

No date, language, or study type limit was applied.

Searches were run on October 26, 2025.

Database(s): **Ovid MEDLINE(R) ALL**
Search Strategy:

#	Searches	Results
1	tocopherols/ or vitamin e/ or alpha-tocopherol/ or gamma-tocopherol/	35277
2	(vitamin e or tocopherol* or tocopheryl*).tw,kf.	51623
3	1 or 2	57633
4	("carboxyethyl hydroxychroman" or CEHC or metabolite*).tw,kf.	397785
5	Urine/	38498
6	Urine Specimen Collection/	653
7	(urine or urinary*).tw,kf.	588452
8	5 or 6 or 7	596699
9	3 and 4 and 8	231

Database(s): **Web of Science**
Search strategy:

#	Searches	Results
1	TS=(("vitamin e" or tocopherol* or tocopheryl*)) AND TS=(("carboxyethyl hydroxychroman" or CEHC or metabolite*)) AND TS=((urine or urinary*))	397

Database(s): **Embase:**
Search strategy:

#	Searches	Results
1	tocopherols/ or vitamin e/ or alpha-tocopherol/ or gamma-tocopherol/	93994
2	(vitamin e or tocopherol* or tocopheryl*).tw,kf.	64816
3	1 or 2	105799
4	("carboxyethyl hydroxychroman" or CEHC or metabolite*).tw,kf.	491615
5	Urine/	214088
6	Urine Specimen Collection/	38093
7	(urine or urinary*).tw,kf.	926083
8	5 or 6 or 7	990874

9	3 and 4 and 8	352

Database(s): **Cochrane**
Search strategy:

**Search ID#**	**Searches**	**Results**
1	(“vitamin e” or tocopherol* or tocopheryl*):ti,ab,kw	9501
2	(“carboxyethyl hydroxychroman” or CEHC* or metabolite*):ti,ab,kw	18159
3	(urine or urinary*):ti,ab,kw	91677
4	#1 AND #2 AND #3	85

Database(s): **CINAHL PLUS:**
Interface - EBSCOhost Research Databases
Search Screen - Advanced Search
Search strategy:

**Search ID#**	**Searches**	**Results**
S1	(MH “vitamin e” or tocopherol* or tocopheryl*)	8,608
S2	(MH “carboxyethyl hydroxychroman” or CEHC* or metabolite*)	23,670
S3	(MH urine or urinary*)	94,368

S4	S1 AND S2 AND S3	37
